# Psychophysical stress during a 24 h dive: A case study of an older male diver

**DOI:** 10.14814/phy2.70836

**Published:** 2026-03-23

**Authors:** Alessandra Barassi, Andrea Brizzolari, Federico Rubino, Anna Caretti, Federica Nenna, Murat Egi, Daniela Caldirola, Alessandra Alciati, Giampaolo Perna, Silvia Daccò, Lara Alessia Moltani, Danilo Cialoni

**Affiliations:** ^1^ Department of Health Sciences Università Degli Studi of Milan Milan Italy; ^2^ ASST Santi Paolo e Carlo Milan Italy; ^3^ Apnea Academy Research Padua Italy; ^4^ Department of General Psychology University of Padua Padua Italy; ^5^ Computer Engineering Department Galatasaray University Istanbul Turkey; ^6^ Department of Biomedical Sciences Humanitas University Como Italy; ^7^ Humanitas, Clinical and Research Center, IRCCS Milan Italy; ^8^ Comftech Srl Monza Italy; ^9^ Department of Human Sciences and Promotion of the Quality of Life San Raffaele University of Rome Rome Italy

**Keywords:** cognitive test, nitric oxide, NT‐proBNP, oxidative stress, scuba diving

## Abstract

This case study investigated the psychophysiological stress responses of a diver during and after a 24‐h hyperbaric exposure. Comprehensive biomarker analysis revealed relevant changes in oxidative stress parameters and cardiac muscle markers. Notably, a critical time point at 6 h (T2) emerged, characterized by peak oxidative stress and diminished antioxidant capacity, followed by progressive cardiac strain as evidenced by elevated creatine kinase isoenzime (CK‐MBm) and N‐terminal prohormone of brain natriuretic peptide (NT‐proBNP) levels. Despite these physiological challenges, no pathological tissue damage was detected, indicating effective endogenous defense mechanisms. Moreover, no significant psychophysiological symptoms were detected before, during, and after the dive, except for tiredness and insomnia. Cognitive assessments reflected adaptive shifts in response strategies under prolonged stress, emphasizing the importance of monitoring to prevent operational risks. These findings highlight the need to consider dive duration carefully, suggesting that introducing rest intervals or personnel rotation around the critical T2 threshold may mitigate physiological fatigue and optimize performance. This study provides valuable insights into human adaptation to extended hyperbaric conditions and informs safety protocols for commercial and military diving operations.

## 1 INTRODUCTION

Self‐Contained Underwater Breathing Apparatus (SCUBA) diving involves multiple physiological stressors, including increased environmental pressure, breathing gas density, hyperoxia, respiratory constraints, physical effort, and cold exposure (Pendergast et al., 2015). These challenges are particularly evident in professional divers, including military, commercial, and technical divers, who are exposed to hyperbaric environments for extended periods (Florian et al., 2016).

Long‐duration dives may require specific breathing mixtures, such as enriched air nitrox, Trimix, or 100% O_2_, to reduce nitrogen narcosis and decompression time. Furthermore, breathing 100% O_2_ at decompression stops increases arterial O_2_ pressure and promotes nitrogen off‐gassing, limits bubble formation, which is one of the necessary factors for the development of decompression sickness (DCS) (Brubakk & Neuman, 2003; Segrt Ribicic et al., 2019).

Prolonged hyperbaric exposure can increase the production of reactive oxygen and nitrogen species (RONS), potentially damaging DNA, lipids, and proteins, and altering immune and vascular function (Levenez et al., 2022; Radojevic‐Popovic et al., 2015). To assess the balance between oxidative stress and antioxidant defense, specific biomarkers can be used, including total antioxidant capacity (TAC) (Cialoni et al., 2019; Cialoni et al., 2020), glutathionyl hemoglobin (HbSSG) (Rubino, 2021), and uric acid, which collectively represent systemic antioxidant defenses, intra‐erythrocyte redox status, and metabolic antioxidant buffering, respectively.

A similar line of reasoning can be applied to variations of nitric oxide (NO) that is a pivotal molecule responsible for the maintenance of ventilation, relaxing the smooth muscles to open the blood vessels (Weinberger et al., 1999) in diving activities (Theunissen et al., 2013).

Extended dives also impose physical and cardiovascular stress, reflected in changes of skeletal muscle markers, including creatine kinase (CK), aspartate transferase (AST), alanine transferase (ALT), and lactate dehydrogenase (LDH) (Cialoni et al., 2021), and in cardiac biomarkers such as creatine kinase isoenzyme (CK‐MBm), N‐terminal prohormone of brain natriuretic peptide (NT‐proBNP), and troponin I (cTnI) (Zarak et al., 2020), (Gempp et al., 2005; Passino et al., 2011).

As reported by several authors, long duration dives reduce lung functions including spirometry parameters (Shykoff & Florian, 2018) and exhaled NO fraction (FeNO) (Maziak et al., 1998), while surfactant proteins (especially SP‐B) may be used to study alveolar stress (Guazzi et al., 2025; Pascual‐Figal et al., 2009).

Cognitive performance may also be challenged due to inert gas narcosis (Abraini, 1997), environmental stressors (Vaernes & Darragh, 1982), and prolonged attentional demands (De Moja et al., 1987). The aim of this study is to investigate vascular, oxidative, muscular, cardiac, pulmonary, and cognitive responses before, during, and after a 24‐h dive, addressing gaps in understanding the integrated physiological and antioxidant adaptations to prolonged hyperbaric exposure. The subject is a 68‐year‐old professional diver who remains highly active, spending many hours underwater each day for work. We hypothesize that long‐duration diving will elicit measurable oxidative and vascular responses, as well as transient alterations in muscular, cardiac, pulmonary, and cognitive function.

## 2 MATERIALS AND METHODS

This case report is presented in accordance with the CARE guidelines (see Supporting CARE checklist S1).

### 2.1 Study design

A 68‐year‐old diver was studied while staying at a depth of 10 meters for 24 continuous hours in the Mediterranean Sea (Spotorno, Italy), wearing a 5 mm wetsuit. (Figure S1A).

He breathed air for 22.5 h and 100% oxygen until the 24‐h mark. The diver has always remained within a radius of 5–6 m from a diving bell placed at the same depth. He went into the diving bell only to eat, hydrate, and undergo scientific tests and blood samples taken during the dive. The instruments (spirometer and FeNO analyzer), what is needed for blood sampling and the questionnaire were brought into the diving bell using a dry bag. Data were collected as follows:

- T0: 30 min before the dive.
- T1: 2 h from dive beginning.
- T2: 6 h from dive beginning.
- T3: 6 h before surfacing (18 h).
- T4: 2 h before surfacing (22 h).
- T5: 30 min after surfacing.

### 2.2 Subject description

The subject of this case report was a healthy 68‐year‐old male commercial diver (height: 185 cm; weight: 92 kg; BMI: 26.9). He was a nonsmoker and on daily antihypertensive therapy (Zanedip 10 mg and Plaunac 20 mg). No history of DCS, or relevant medical conditions, was reported. The study was conducted in accordance with the Helsinki Declaration, approved by the Ethical Committee of Università degli Studi di Milano (Aut. no. 37/17). The subject provided written informed consent.

### 2.3 Blood and vascular‐endothelial stress

Whole blood samples (WBS) were analyzed within 48 h (Unalli & Ozarda, 2021) using a Medonic M20 hemocromocytometer (Boule, Spånga, Sweden) to measure the following hematological parameters: white blood cells (WBC), lymphocytes (LYM), granulocytes (GRAN), hemoglobin (HGT), mean corpuscular hemoglobin (MCH), mean corpuscular hemoglobin concentration (MCHC), red blood cells (RBC), mean corpuscular volume (MCV), hematocrit (HCT), red cell distribution width (RDW), platelets (PLT), and mean platelet volume (MPV).

Plasma NO derivatives (NOx) were determined in the deproteinized plasma. Standard solutions of NaNO_3_ in a concentration range from 5 to 200 μM were used to build the nitrate (NO_3_) calibration curve. NOx was determined according to Brizzolari et al. (Brizzolari et al., 2021).

### 2.4 Oxidative stress

Total antioxidant capacity (TAC) was investigated using Trolox Equivalent Antioxidant Capacity (TEAC) assay (Re et al., 1999), according to Cialoni et al. (Cialoni et al., 2020).

Serum uric acid was measured using an automatic VITROS® 5600 analyzer (Ortho‐Clinical Diagnostics, High Wycombe, United Kingdom), although uric acid levels are influenced by multiple metabolic and renal factors and therefore do not represent a unique, specific marker of antioxidant defense.

Gluthathionyl Hemoglobin (HbSSG) was measured in RBC with a published original method that employs MALDI‐ToF mass spectrometry (Rubino et al., 2023).

### 2.5 Cardiomuscular stress

Serum CK, CK‐MBm, NT‐proBNP, AST, ALT, CRP, and cTnI were analyzed as previously described (Cialoni et al., 2021). Echocardiography before and after the dive was performed to detect circulating bubbles (Eftedal & Brubakk, 1993). Continuous ECG, respiratory rate, posture, and SpO_2_ were recorded via a sensorized wearable T‐shirt (Comftech srl, Monza, Italy) to monitor cardiovascular and muscular stress in real time.

### 2.6 Lung stress

Lung function was evaluated by spirometry (VC, FVC, FEV1, FEV1/FVC, FEF25–75) using a Spirobank II spirometer (MIR, Berlin, Wisconsin USA) and FeNO using a FeNO Breath (Bedfont Scientific, Maidstone, UK). Ultrasound lung comets (ULCs) (Frassi et al., 2008) and plasma surfactant‐B (SP‐B) were measured to assess pulmonary response (Dei Cas et al., 2025).

### 2.7 Psychological stress and cognitive function

Physical symptoms, emotional responses, and sleep quality were assessed using VAS, RPE, POMS, PSL, BVS, D‐12, FQ, ISI (McNair & Droppleman, 1971), and BORG scales (Borg, 1982). Cognitive performance was evaluated using an underwater Go/No‐Go task (Gomez et al., 2007) with dual visual cues, recording reaction times and accuracy to monitor attention, inhibitory control, and adaptability under hyperbaric conditions.

### 2.8 Analytical performance

All biochemical and hematological assays were performed in duplicate. Intra‐assay coefficients of variation (CVs) were below 20% for SP‐B, 10% for total antioxidant capacity (TAC), and below 5% for whole blood analysis, serum uric acid, TBARS, and serum cardiomuscular biomarkers.

Measurements performed using medical equipment were calibrated prior to use according to the manufacturers' instructions.

For nitrate/nitrite (NOx) determinations, analytical performance was as previously reported by Brizzolari et al. (Brizzolari et al., 2021). For HbSSG determinations, analytical performance was as previously reported by Rubino et al. (Rubino et al., 2023).

## 3 RESULTS

The diver completed the 24‐h dive without health issues. About an hour postdive, he experienced brief malaise and a 15‐s loss of consciousness, likely due to fatigue. Medical personnel assisted immediately; arrhythmias recorded resolved spontaneously within minutes. Dive parameters: maximum depth 10 m, average 9 m, water temperature 24°C, 34,000 L air and 2000 L 100% O_2_ consumed.

### 3.1 Blood and vascular‐endothelial stress

**FIGURE 1:**
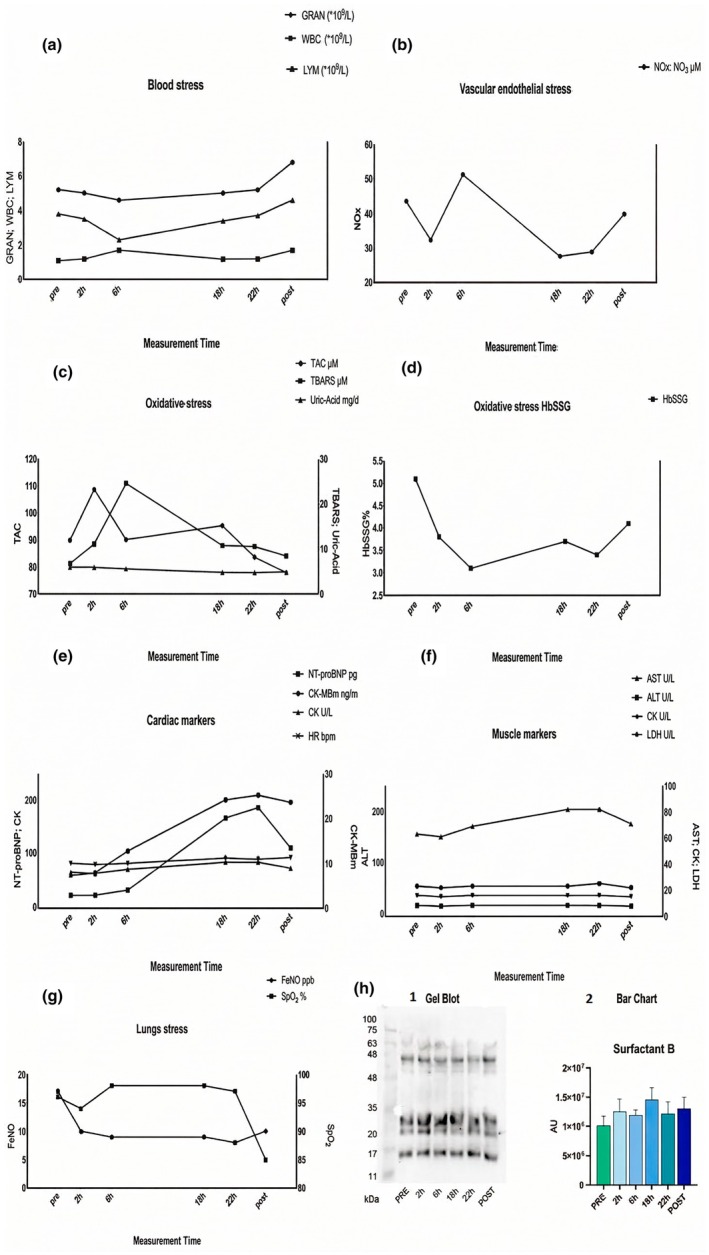
Overview of physiological and biochemical parameters investigated. (a) Peripheral blood cell count, (b) vascular‐endothelial stress, (c) TAC and TBARS, (d) Glutathionyl hemoglobin (HbSSG), (e) cardiac markers, (f) muscle markers, (g) lung's stress, (h) circulating SP‐B expression determined by Western Blotting. Data are presented as mean ± SD.

WBC and GRAN levels showed a slight decrease at T2, followed by an increase after the dive while LYM levels increased at T2, then decreased during the dive, and rose again after surfacing (Figure 1a).

Also, RBC, HGB, and HCT levels showed a slight decrease at 6 h (T2), stabilizing afterward. PLT levels decreased from predive to T2. The other parameters remained stable throughout the dive. Regarding NOx, we observed a decrease in values, except at T2, where a sudden increase was noted (Figure 1b).

Table 1 reports a detailed trend of each e blood count parameter and NOx changes.

**TABLE 1:** Blood count parameters.

Parameter	T0	T1	T2	T3	T4	T5
WBC (×10^9^/L)	5.2	5	4.6	5	5.2	6.8
LYM (×10^9^/L)	1.1	1.2	1.7	1.2	1.2	1.7
GRAN (×10^9^/L)	3.8	3.5	2.3	3.4	3.7	4.6
MCH (pg)	30.3	30.4	30.5	30.9	30.9	30.5
MCHC (g/L)	35.1	35.1	34.7	36.2	35.9	35.8
RBC (×10^12^/L)	4.4	4.17	3.62	4.53	4.42	4.7
MCV (fL)	86.3	86.5	87.7	85.5	85.9	85
HGB (g/L)	13.3	12.7	11	14	13.6	14.3
HCT (%)	38	36.1	31.7	38	37.8	40
RDW (%)	14.4	14.2	14.6	14.3	14.5	14.1
PLT (×10^9^/L)	225	194	163	181	184	208
MPV (fL)	8.1	7.9	9.6	7.8	7.9	8.3
Vascular‐endothelial stress						
NOx (μM NO_3_)	43.5	32.4	51.3	27.6	28.8	39.8

### 3.2 Oxidative stress

As for oxidative stress parameters (Figure 1c), TAC values rose at T1, then gradually declined until the end of the dive and continued to decrease after surfacing. TBARS rose until T2 showing its peak, then decreased coming back to values similar to those pre diving. Uric acid levels showed a reduction starting from T2 during the dive. HbSSG showed a decrease during the dive, with respect to the predive value, and a still partial return to predive value after resurfacing. The maximum decrease was at T2 (−39.2%) during the diving period (Figure 1d).

Table S1A in Supporting Information reports a detailed trend of NOx level and each oxidative stress parameter.

### 3.3 Cardiomuscular stress

Figure 1e showed NT‐proBNP and CK‐MBm progressively rise up from 6 h to 18–22 h, whereas in Figure 1f, muscle markers showed relatively stable values for AST, ALT, and LDH.

Physiological heart parameters did not show any significant variations throughout the 24‐h diving protocol. This suggests that, despite the environmental and physical challenges imposed by the 24‐h dive, basic physiological parameters remained stable. These findings may reflect a good level of adaptation in trained subjects and confirm the feasibility and reliability of wearable technology for continuous monitoring in extreme conditions. No differences were noted in the formation of bubbles.

Viewed detail in Table S1B in Supporting Information.

### 3.4 Lung stress

**FIGURE 2:**
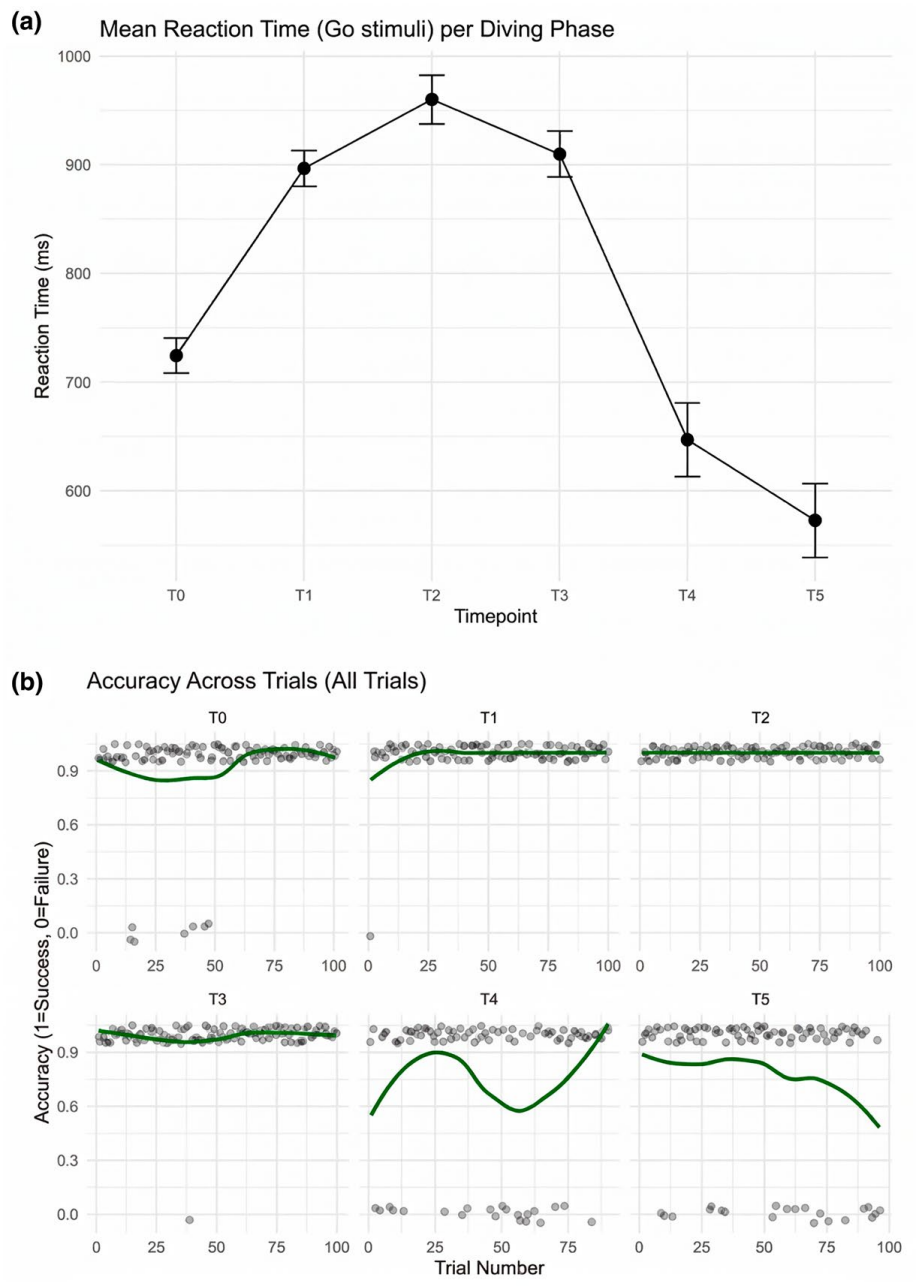
Go‐NoGo test reaction times at the Go stimuli.

Spirometry measurements showed no notable changes during or after the dive, while ultrasound lung comets increased after surfacing. As shown in Figure 1g, FeNO levels decreased 2 h after dive start and remained stable thereafter. SpO_2_ decreased postdive, remained stable during the dive, and dropped sharply upon exit. Circulating SP‐B, a biomarker of alveolar damage, showed a slight overall increase, peaking at T3 (Figure 1h‐1,h‐2). Table S1C in Supporting Information reports instrumental values indicating lung stress.

### 3.5 Psychological stress and cognitive functions

Table 2 reports the psychometric assessment scores.

**TABLE 2:** Psychometric assessment scores.

	Timing					
	T0	T1	T2	T3	T4	T5
Visual Analog Scale, cm						
Wellness/discomfort	0	0	0	0	3	3
Restfulness/tiredness	0	0	0	0	**5**	**10**
Hot/cold	4	0	0	0	0	2
Trembling or shaking	0	0	0	0	0	0
Headache	0	0	0	0	0	0
Nausea	0	0	0	0	0	0
Happiness/unhappiness	0	0	0	0	2	2
Sensation of constriction/oppression/lack of movement freedom	0	0	0	0	0	0
Dizziness	0	0	0	0	0	0
Derealization	0	0	0	0	0	0
Level of fear	0	0	0	0	0	0
Shortness of breath	0	0	0	0	0	0
Tachycardia	0	0	0	0	0	0
Profile of mood states						
Tension‐anxiety (range 0–36)	2	_	_	_	_	0
Depression‐dejection (range 0–60)	0	_	_	_	_	0
Anger‐hostility (range 0–48)	0	_	_	_	_	1
Vigor‐activity (range 0–32)	20	_	_	_	_	5
Fatigue‐inertia (range 0–28)	2	_	_	_	_	2
Confusion‐bewilderment (range 0–28)	0	_	_	_	_	0
Panic symptoms list‐based discomforts (range 0–13)	1^a^	_	_	_	_	0
Dyspnea‐12 (range 0–36)	0	_	_	_	_	0
Body vigilance scale (range 0–31)	12.5	_	_	_	_	_
Fear questionnaire						
Agoraphobia (range 0–40)	1^~^	_	_	_	_	_
Blood–injury phobia (range 0–40)		_	_	_	_	_
Social phobia (range 0–40)		_	_	_	_	_
FQ total score (range 0–120)	1	_	_	_	_	_
Insomnia Severity Index (range 0–28)	0	_	_	_	_	_
Sleep attitude during the experiment*						
Difficulty falling asleep	Extremely**					
Difficulty staying asleep	Extremely**					
How satisfied/dissatisfied are you with your current sleep?	Not at all satisfied***					
How much time did you sleep during the experiment? (minutes)	0					

*Note*: h, hours from the dive starting point. *Insomnia Severity Index, modified ad hoc items to evaluate the sleep attitude during the experiment (**5‐point Likert scale: Not at all to Extremely; ***5‐point Likert scale: Not at all satisfied to Extremely satisfied).

^a^ The subject reported the presence of sweating; ~ the subject reported slight avoidance in “walking alone in crowded streets” item.

The only consistent observations were a high level of tiredness following the experiment and total absence of sleep; finally, the BORG scale score exhibited the highest value, 20.

Cognitive performance showed a variable pattern across the 24‐h dive. Reaction times to Go stimuli (Figure 2a) initially increased at T1 (897 ± 136 ms) and T2 (960 ± 191 ms) compared to baseline (T0: 724 ± 132 ms), followed by a progressive decrease at T4 (647 ± 224 ms) and T5 (573 ± 242 ms). While Go accuracy remained high during the first phases of immersion (≥ 90% until T3), a marked decline was observed at T4 (74.6%) and T5 (73.9%). No‐Go accuracy, initially at ceiling levels (100% at T0 and T2), progressively decreased during the dive, reaching 80.6% at T4. In addition to the overall changes in accuracy across time points, the trial‐by‐trial accuracy plot (Figure 2b) revealed further intrasession fluctuations, particularly in the later diving phases. Accuracy remained stable across trials during the early sessions (T0–T3), consistently fluctuating around ceiling levels. However, starting from T4, accuracy showed a higher variability across trials, with alternating periods of preserved and decreased performance emerging within the same session.

It remains to be investigated whether these fluctuations temporally correspond to variations in physiological or psychometric measures collected in parallel throughout the dive.

## 4 DISCUSSION

### 4.1 Blood and vascular endothelial stress

We observed some changes in the hematological values, both during and after the dive, peaked at 6 h (T2); even if all values remained within the range considered, these changes could be related to acute oxidative stress, immune activation, and prolonged physical effort (Galun et al., 1987). The highest value of WBC observed at T5 may also reflect a significant immune response that usually accompanies long duration physical effort (Kakanis et al., 2010).

While circulating lymphocytes increase, likely in response to an acute inflammatory state in the peripheral circulation (Thorley et al., 1977), GRAN blood value decrease probably because they are recruited to potentially inflamed tissues (Summers et al., 2010). Furthermore, LYM increase could be explained as a consequence of the activation of antioxidant defenses in order to protect the cells against the induction of oxidative damage (Vangala et al., 1998) detoxifying free radicals (Ferrer et al., 2007). It is intriguing to note that both of these changes begin immediately at the start of the dive and continue until T2, which seems to represent a critical point in the body's blood response to this extreme dive.

Regarding NOx, we observed an increase in values at T2, likely associated with the initial intense regulation of vasoconstriction and vasodilation mechanisms required for the organism to adapt to the hyperbaric environment (Cialoni et al., 2019; Lundberg et al., 2015). This increase may also be partially attributable to NO oxidative processes (van Vliet et al., 1997).

### 4.2 Oxidative stress

Exposure to hyperbaric environments promotes RONS production and oxidative stress (Mrakic‐Sposta et al., 2024; Perovic et al., 2014). Consequently, endogenous antioxidant defenses are activated to maintain cellular redox balance (Valko et al., 2007) and limit vascular oxidative stress associated with ROS release (Cialoni et al., 2019; Mrakic‐Sposta et al., 2024; Sureda et al., 2012). Accordingly, TAC increased during the initial dive phase (first 2 h), followed by a marked reduction—especially at T2—and a progressive decline, likely due to antioxidant consumption. The onset of TAC decline coincided with the TBARS peak (T2), which then decreased and stabilized during the final dive phase. Regarding HbSSG, the predive level of 5.1% is in the range measured for healthy subjects (Rubino et al., 2023). We found a decrease of values during the dive and a still partial return to predive value after re‐surfacing with a maximum decrease (−40%) at 6 h, so once again at T2. The observation that the level of HbSSG reverts toward preexercise conditions readily after the relatively short postexercise observation time shows that in this old, yet healthy subject the antioxidant resilience mechanism operates efficiently and that the RBC antioxidant enzymes have not been impaired or inactivated during the long diving period, for example, by irreversible oxidation or covalent modification by ROS and reactive electrophiles, such as those measured as TBARS. The time trend of HbSSG during the diving challenge shows elicitation of the antioxidant response to breathing an oxygen‐enriched atmosphere at over‐ambient pressure. In fact, the transient increase of NOx and TBARS levels at T2 would not cause an increase of HbSSG in this subject, although NOx is a recognized inducer of HbSSG production [cit] and several TBARS are able to inactivate antioxidant enzymes. Evidence on the effect of underwater diving under different conditions on HbSSG levels is still scanty. However, bi‐modal response is by no means limited to strong physiological challenge from diving, but also to other extreme including months‐long stay in Antarctica at hypoxic oxygen levels (Rigamonti et al., 2024), with a similar outcome.

On the other hand, it should be acknowledged that the antioxidant‐related markers assessed in the present study are indicative but not definitive of the overall antioxidant status, particularly due to the absence of data on enzymatic antioxidant systems.

### 4.3 Cardiomuscular stress

Cardiac biomarkers (CK, CK‐MBm, and NT‐proBNP) increased during the dive, reflecting skeletal and cardiac workload without signs of organ injury, as indicated by stable AST, LDH, ALT, and ECG. CK‐MBm, specific to cardiac muscle, showed a progressive increase from predive values, paralleled by NT‐proBNP. The rise in CK‐MBm may result from prolonged physical effort (Siegel et al., 1983) and atrial stretching (Theunissen et al., 2013). According to several authors (Gempp et al., 2005; Grassi et al., 2007; Passino et al., 2011), increased NT‐proBNP may be due to cardiomyocyte stretch induced by hyperbaric‐related plasma volume changes, blood shift, and atrial pressure variations, leading to cardiac distension and increased stroke volume and cardiac output (Gabrielsen et al., 1993; Lippi et al., 2008).

### 4.4 Lung stress

Regarding lung response, spirometry parameters showed no differences pre‐, during, or postdive, suggesting no effect of diving. Ultrasound comets after surfacing appeared related to physical exertion, though only six were observed, within the normal range. FeNO, while remaining within normal values (5–25 ppb), decreased during and after the dive compared to pre‐dive, likely due to inhibition of inducible NO synthase (iNOS) by hyperbaric hyperoxia (Puthucheary et al., 2006) or improved ventilation–perfusion, enhancing NO diffusion (Pendergast & Lundgren, 2009). The lack of FeNO increase indicates that prolonged hyperbaric exposure did not trigger pulmonary inflammation.

It is important to emphasize that FeNO primarily reflects nitric oxide production in the airways, being influenced by pulmonary epithelial activity, ventilation‐perfusion dynamics, and other local factors. It is important to note that FeNO is not directly related to circulating NOx levels, which represent systemic NO production and metabolism across multiple tissues and are not specific to vascular endothelial function. Therefore, FeNO should be interpreted as a marker of pulmonary NO activity.

The postdive reduction in SpO_2_ may be attributable to inert gas accumulation combined with the sustained physical workload imposed by the prolonged dive. This alteration could also account for the diver's acute episode of malaise, plausibly related to fatigue and transient cerebral hypoxia, as indicated by the brief loss of consciousness.

### 4.5 Psychological stress and cognitive performance

The psychometric assessment showed that the diver experienced no specific physical symptoms or emotional reactions during performance, consistent with previous research on well‐trained individuals (Sarkar & Fletcher, 2014). A transition from restfulness to tiredness was observed at 22 h, peaking at the experiment's end, as indicated by post‐assessment VAS scores. This shift reflects prolonged physical effort, evidenced by maximal BORG scores, and lack of sleep, aligning with studies on the cumulative effects of exertion and sleep deprivation on fatigue perception (Kong et al., 2025). Go/noGo trends suggest the occurrence of cognitive fluctuations throughout the prolonged dive period. The intra‐session fluctuations suggest possibly occurring transient lapses of attention or instability in response control during prolonged underwater exposure.

### 4.6 Limitation

It is important to acknowledge that this study reports findings from a single‐case design, which inherently limits the generalizability of the results. Although the data provide valuable insights into physiological and psychophysiological responses during prolonged hyperbaric exposure, caution should be exercised when extrapolating these observations to larger populations. It should be noted that several factors beyond our experimental control may have influenced the observed hematological and oxidative responses. Variations in hydration status, oxygen exposure, or environmental conditions could have contributed to changes in blood cell counts, oxidative stress markers, and cardiovascular parameters. Additionally, lifestyle factors or diet may have affected the physiological responses. While these variables cannot be fully excluded, the overall trends remain consistent with an adaptive response to prolonged hyperbaric exposure. Future studies with controlled interventions are needed to clarify the relative contribution of these potential confounders.

## 5 CONCLUSIONS

A 24‐h hyperbaric exposure induces reversible physiological stress, characterized by oxidative, cardiac, and fatigue‐related changes, without tissue damage. Biomarker changes highlight 6 h as a critical phase, relevant for dive planning and monitoring, informing human adaptation and safety in prolonged dives.

## AUTHOR CONTRIBUTIONS

Conceived and designed research: ABr and DCi; performed experiments: ABa, FMR, AC, and ABr; analyzed data: ABr, FMR, AC, FN, AA, DCa, SD, and DCi; interpreted results of experiments: ABr, FMR, AC, FN, AA, DCa, SD, and DCi; prepared figures Abr, FMR, AC, FN and DCi; drafted manuscript: ABr; edited and revised manuscript: FMR, AC, FN, ME, AA, DCa, GP, SD, and DCi; approved final version of manuscript: ABr and DCi. The information must be the same as in the online submission site.

## FUNDING INFORMATION

No funding external to the University of Milan and University S. Raffaele of Rome was received.

## CONFLICT OF INTEREST STATEMENT

No conflict of interest, financial, or otherwise is declared by the authors.

## Supporting information


Data S1.

Data S2.

## Data Availability

The data that support the findings of this study are available from the corresponding author upon reasonable request.
