# Supplementary material for: Psychophysical stress during a 24 h dive: A case study of an older male diver
Source: Physiol Rep. 2026 Mar 23;14(6):e70836. doi: 10.14814/phy2.70836 (PMC13098038; doi:10.14814/phy2.70836)
Supplement: Supplementary file 1 — Data S1. [file PHY2-14-e70836-s002.docx]

**Supplementary Material**

**Supplementary Figure A - Experimental protocol, instrumental tests, sample collection and scale administration plan.**


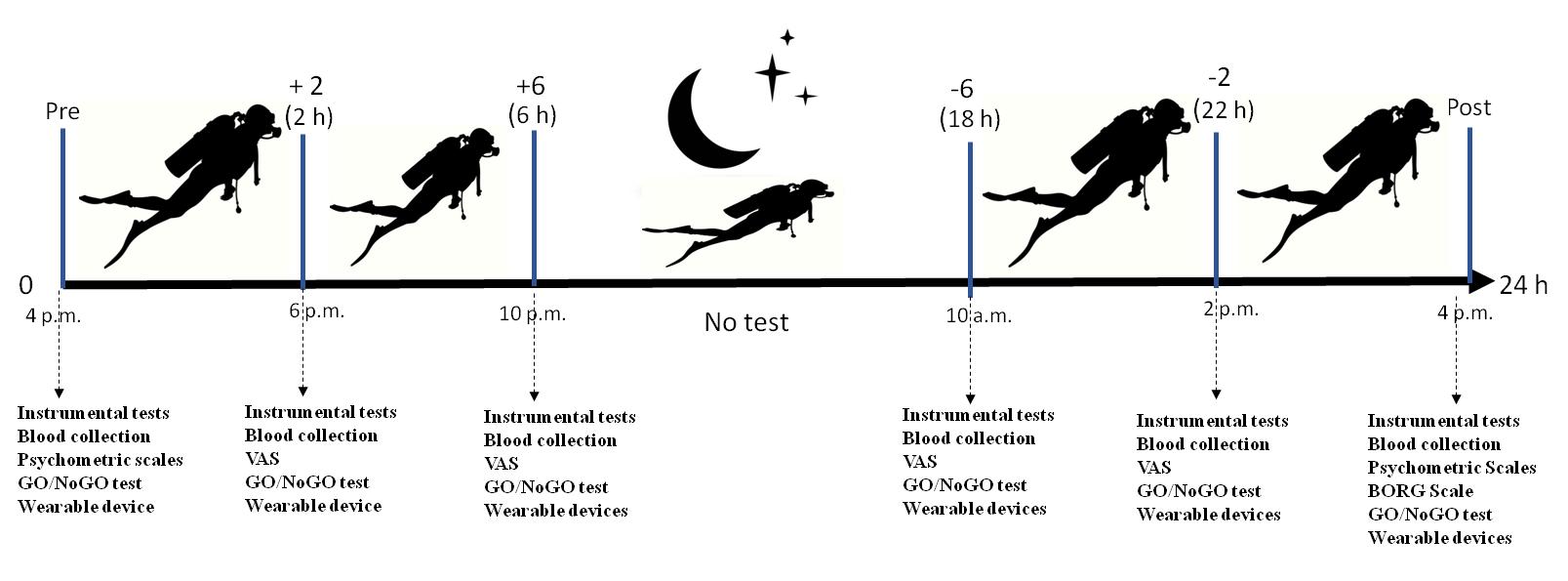


**Supplementary Table A – Endothelial and Oxidative stress parameters.**

| **Parameter** | **T0** | | **T1** | | **T2** | **T3** | | **T4** | **T5** |
| --- | --- | --- | --- | --- | --- | --- | --- | --- | --- |
| TAC (µM Trolox eq.) | | 89.9 | 108.7 | 90.1 | | 95.3 | 83.7 | | 77.7 |
| Uric Acid (mg/dL) | | 5.9 | 5.9 | 5.5 | | 4.8 | 4.7 | | 4.9 |
| TBARS (µmol MA/L) | | 6.7 | 11.1 | 24.6 | | 10.7 | 10.6 | | 8.4 |
| HbSSG (%) | | 5,1% | 3,8% | 3,1% | | 3,7% | 3,4% | | 4,1% |

**Supplementary Table B – Cardiomuscular biomarkers of stress.**

| **Parameter** | **T0** | **T1** | **T2** | **T3** | **T4** | **T5** |
| --- | --- | --- | --- | --- | --- | --- |
| cTnI (ng/ml) | 0.012 | 0.012 | 0.012 | 0.012 | 0.012 | 0.012 |
| CK (U/L) | 63 | 61 | 69 | 82 | 82 | 71 |
| CK-MBm (ng/ml) | 0.35 | 0.39 | 1.21 | 1.26 | 1.26 | 1.18 |
| NT-proBNP (pg/ml) | 20 | 20 | 29.7 | 166 | 186 | 209 |
| AST (U/L) | 23 | 22 | 23 | 23 | 25 | 22 |
| ALT (U/L) | 16 | 15 | 16 | 16 | 16 | 15 |
| LDH (U/L) | 135 | 130 | 133 | 147 | 168 | 172 |
| CRP (mg/L) | 5 | 5 | 5 | 5 | 5 | 5 |
| Heart Rate (HR) | 80 | 78 | 80 | 90 | 88 | 91 |
| Bubble (n) | 0 | - | - | - | - | 0 |

**Supplementary Table C – Instrumental measurements.**

| **Spirometry** | | | | | | |
| --- | --- | --- | --- | --- | --- | --- |
| **Parameter** | **T0** | **T1** | **T2** | **T3** | **T4** | **T5** |
| VC | 4,74 | 5,55 | 5,6 | 5,81 | 5,51 | 5,76 |
| FVC | 6,09 | 5,8 | 5,7 | 5,55 | 5,7 | 6,11 |
| FEV1 | 4,33 | 4,06 | 4,1 | 3,78 | 4,01 | 4,31 |
| FEV1/FVC (%) | 71,1 | 70 | 71 | 68,1 | 70,6 | 70,5 |
| PEF | 11,2 | 10,57 | 10,1 | 8,66 | 8,83 | 12,33 |
| FEF2575 | 2,83 | 2,63 | 2,8 | 2,33 | 2,62 | 2,81 |
| FEV1/FEV6 (%) | 72 | 71,4 | 72,3 | 69,5 | 71,7 | 71,6 |
| FEV1/VC (75) | 78 | 73,2 | 72,5 | 65,1 | 69 | 74,2 |
| **Other measurements** | | | | | | |
| FeNO (ppb) | 17 | 10 | 9 | 9 | 8 | 10 |
| Ultrasound Lung Comet (n) | 0 | - | - | - | - | 6 |
| SpO_2_ (%) | 96 | 94 | 98 | 98 | 97 | 85 |
