# Supplementary material for: Psychophysical stress during a 24 h dive: A case study of an older male diver
Source: Physiol Rep. 2026 Mar 23;14(6):e70836. doi: 10.14814/phy2.70836 (PMC13098038; doi:10.14814/phy2.70836)
Supplement: Supplementary file 2 — Data S2. [file PHY2-14-e70836-s001.docx]

# CARE Checklist (2016)

| **Topic** |  | **Checklist Item Description** | **Line/page** |
| --- | --- | --- | --- |
| Title | 1 | Yes – the title includes the words “case report” and specifies the area of focus. **Psychophysical stress during a 24 h dive:**  ***a case study of an older male diver*** | Pag. 1 |
| Keywords | 2 | Yes – six keywords are provided, including “case report”. oxidative stress, nitric oxide, scuba diving, NT-proBNP, cognitive test, case report | Pag. 2 |
| Abstract | 3a | This case report adds novel insight into human psychophysiological adaptation to prolonged hyperbaric exposure by identifying a critical 6-hour time point of peak oxidative stress and cardiac strain, highlighting implications for safety protocols in commercial and military diving. | Pag. 1/2 |
| Abstract | 3b | Yes – the abstract summarizes the case and reports outcomes. We found transient increases in oxidative stress and cardiac biomarkers without evidence of pathological tissue damage, alongside preserved psychophysiological functioning. | Pag. 1/2 |
| Abstract | 3c | Yes – the abstract conclusion provides a clear take-away message with practical implications for monitoring during prolonged hyperbaric exposure. | Pag. 1/2 |
| Introduction | 4 | Yes – the introduction summarizes current knowledge on prolonged hyperbaric exposure and explains the contribution of this case report to the available literature, with appropriate references. | Pag. 2/3 |
| Timeline | 5 | Yes – a chronological timeline of exposure and measurements is provided in Figure 1 describing the experimental protocol. | Pag. 3 |
| Participant Information | 6a | Yes – de-identified participant characteristics are reported at the beginning of the Materials and Methods section.  The subject of this case report was a healthy 68-year-old male commercial diver (height: 185 cm; weight: 92 kg; BMI: 26.9). | Pag. 3 |
| Participant Information | 6b | Yes – de-identified participant characteristics are reported at the beginning of the Materials and Methods section.  He was a non-smoker and on daily antihypertensive therapy (Zanedip 10 mg and Plaunac 20 mg) | Pag. 3 |
| Participant Information | 6c | Yes – the participant has no history of relevant pathologies or prior interventions. This information is reported in the Materials and Methods section. | Pag. 3 |
| Physical Findings | 7 | Yes – relevant physical findings including baseline vital signs and clinical observations during the study are reported in the Materials and Methods section and Results | Pag. 3-11 |
| Diagnostic Assessment | 8a | Yes – investigations including biomarker analysis, psychophysiological assessments, cognitive testing, and devices used for data collection are reported in the Methods section and Results. | Pag. 3-11 |
| Diagnostic Assessment | 8b | Not applicable – the participant was healthy, and no alternative diagnoses or diagnostic challenges were present. | N/A |
| Diagnostic Assessment | 8c | Yes – three figures and two tables, along with additional supplementary tables referenced in the text, are provided to support comprehension of assessments | Pag. 3-7-8-10 |
| Diagnostic Assessment | 8d | Not applicable – the participant was healthy and no prognostic characteristics were relevant. | N/A |
| Intervention | 9a | Not applicable – no therapeutic interventions were performed. | N/A – no therapeutic intervention |
| Intervention | 9b | Not applicable – no therapeutic interventions were performed. | N/A |
| Intervention | 9c | Not applicable – no therapeutic interventions were performed | N/A |
| Intervention | 9d | Not applicable – no other concurrent interventions were administered during the study period. | N/A |
| Follow-up and Outcomes | 10a | Yes – follow-up assessments were conducted throughout the 24-hour hyperbaric exposure, including during immersion, and post-exposure; outcomes included transient biomarker changes and preserved psychophysiological functioning. | Pag. 6-11 |
| Follow-up and Outcomes | 10b | Yes – the study protocol was completed as planned, with no significant adverse events. Only a brief episode of dizziness after the final tests, which resolved within a few seconds. | Pag. 6-11 |
| Follow-up and Outcomes | 10c | Yes – the participant tolerated the study well; the extreme environmental conditions were more stressful than the test protocol itself. | Pag. 6-11 |
| Discussion | 11a | Yes - the strengths of the study include detailed physiological and psychophysiological monitoring during prolonged hyperbaric exposure, providing valuable insights. Limitations include the single-case design, which restricts generalizability, and potential confounding factors such as hydration, oxygen exposure, environmental conditions, and lifestyle. | Pag. 12-14 |
| Discussion | 11b | This case report informs practice by highlighting the importance of monitoring physiological and psychophysiological responses during prolonged hyperbaric exposure, identifying a critical threshold for fatigue, and suggesting operational strategies such as rest intervals or personnel rotation to optimize safety and performance. While based on a single case, these findings can suggest for guide safety protocols and inform future Clinical Practice Guidelines in extreme environmental conditions. | Pag. 12-14 |
| Discussion | 11c | This case report suggests a testable hypothesis that monitoring physiological and psychophysiological responses at specific time points during prolonged hyperbaric exposure can identify critical thresholds of fatigue and oxidative stress, and that interventions such as scheduled rest intervals or personnel rotation may mitigate these effects. Future controlled studies can evaluate these strategies in larger populations. | Pag. 12-14 |
| Discussion | 11d | This case demonstrates that prolonged hyperbaric exposure induces transient physiological stress without causing tissue damage, emphasizing the importance of continuous monitoring and operational strategies to ensure safety. The findings provide valuable insights into human adaptation in extreme environments and inform future protocols and research. | Pag. 12-14 |
| Participant Perspective | 12 | Yes – the participant was assessed before, during, and after the 24-hour hyperbaric exposure, including physiological, psychophysiological, and cognitive evaluations, as detailed in Figures 1–3 and Tables 1–2 (including supplementary materials). | Pag. 3-7-8-10 |
| Informed Consent | 13 | Yes – written informed consent was obtained from the participant, as stated in the Ethics Statement section. | Pag 4 |
| Additional Information |  | Acknowledgements, Competing Interests, and Ethics approval are provided in the manuscript | Pag 15 |
